# Supplementary figures and images for: Viral Metagenomic Data Analyses of Five New World Bat Species from Argentina: Identification of 35 Novel DNA Viruses
Source: Microorganisms. 2022 Jan 24;10(2):266. doi: 10.3390/microorganisms10020266 (PMC8880087; doi:10.3390/microorganisms10020266)

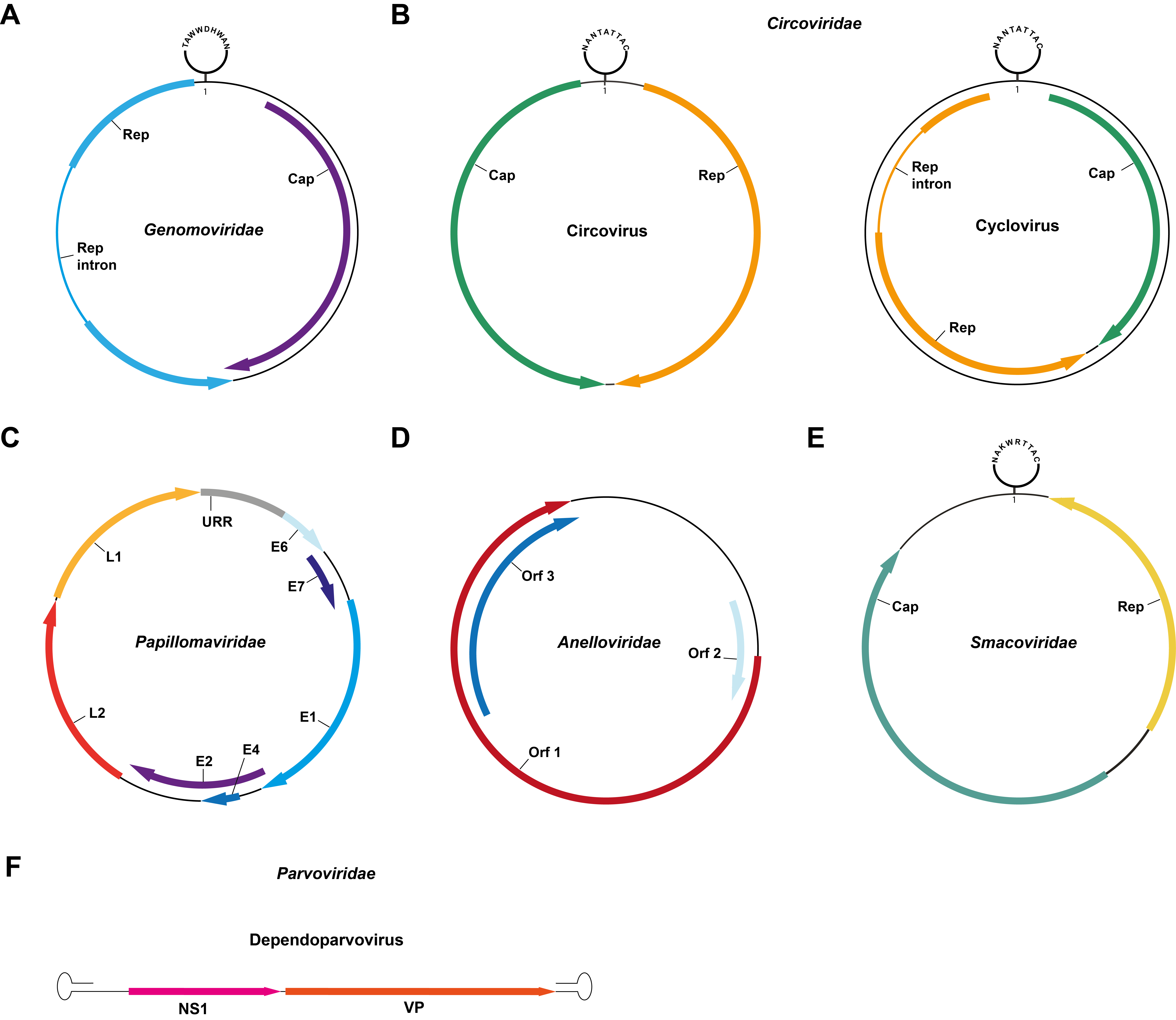

Supplement: Supplementary file 1 [file microorganisms-10-00266-s001.zip › Figure S2.png]

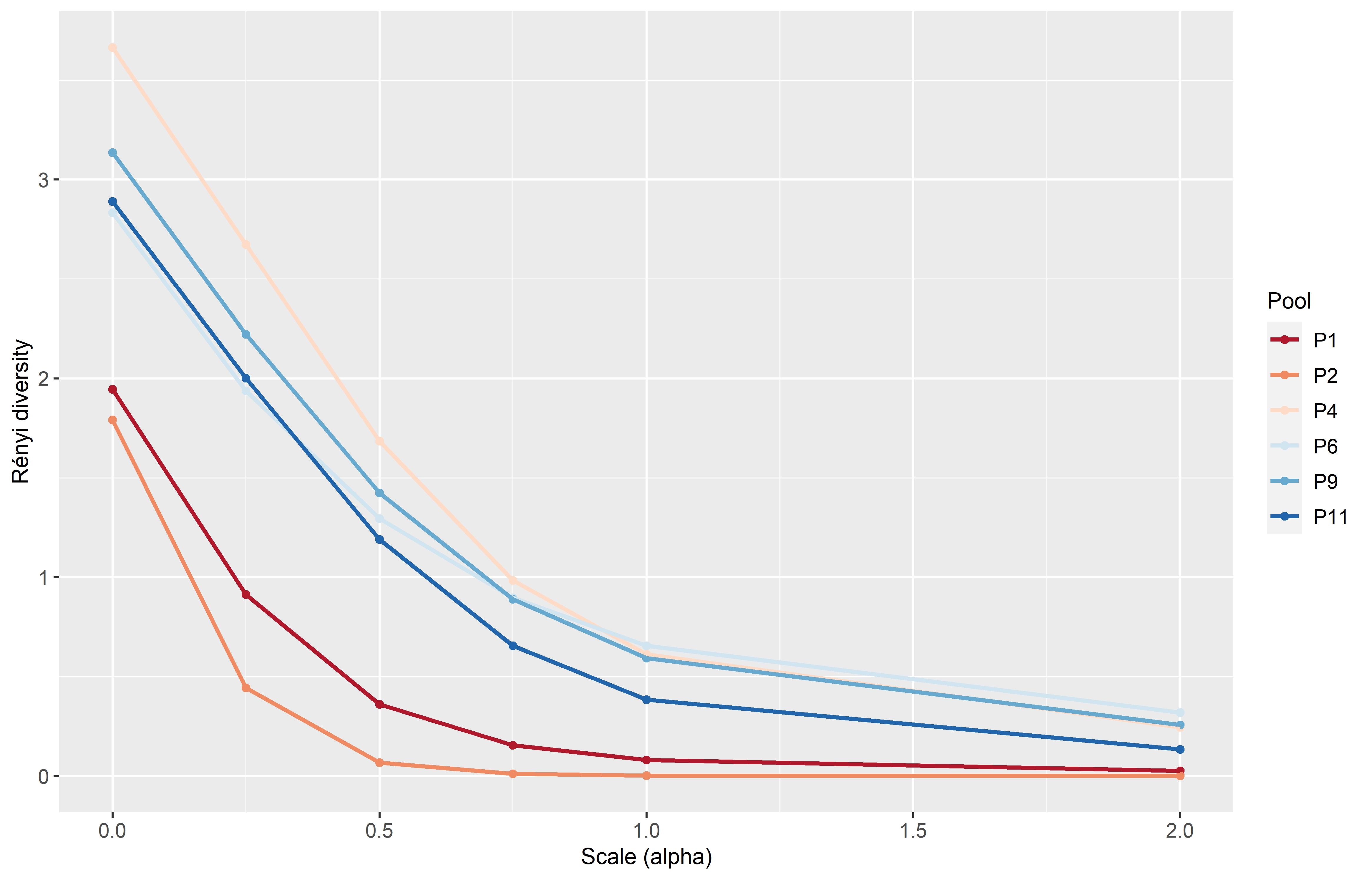

Supplement: Supplementary file 1 [file microorganisms-10-00266-s001.zip › Figure S1.jpg]
